# A semantic strategy instruction intervention aimed at boosting young and older adults’ visual working memory capacity

**DOI:** 10.3758/s13421-024-01676-8

**Published:** 2025-03-05

**Authors:** Rebecca Hart, Louise A. Brown Nicholls

**Affiliations:** Department of Psychological Sciences and Health, University of Strathclyde, 40 George Street, Glasgow, G1 1QE UK

**Keywords:** Visual working memory · Strategy instruction training · Semantic long-term memory · Cognitive ageing/aging

## Abstract

Greater semantic availability (meaningfulness) within visual stimuli can positively impact visual working memory performance. Across two experiments, we investigated the effects of semantic availability and, for the first time, semantic strategy instruction on visual working memory performance. Experiment 1 focused on young adults’ (aged 18–35 years) strategies during visual matrix task recognition. Results highlighted an existing propensity to report incorporating a semantic strategy. Interestingly, there was no significant effect of semantic availability within the task stimuli. Semantic strategy instruction also did not boost, or indeed hinder, accuracy. Experiment 2 incorporated older adults (aged 60–87 years) and highlighted marked differences in capacity with older age. Greater semantic availability reliably benefitted capacity for young adults only. Furthermore, semantic strategy instruction neither boosted nor hindered capacity, even in older adults. There were also some interesting patterns regarding reported strategy use across groups. Again, participants reported spontaneously using semantic strategies, particularly young adults. However, instruction may have encouraged more frequent use of semantic strategies in older adults. Finally, the results suggest a role for task practice, likely related to strategy development and implementation over time. Future semantic strategy instruction protocols may need to incorporate more extensive training and/or practice to benefit working memory capacity.

**Supplementary information:**

The online version contains supplementary material available at 10.3758/s13421-024-01676-8.

## Introduction

Multiple-component models of working memory comprise two specialized components for storing and processing verbal and visuospatial material (the phonological loop and the visuospatial sketchpad, respectively). The episodic buffer allows conscious access to multimodal representations, and may draw upon long-term memory (Baddeley, 2007, 2012; Logie, 2011, 2016, 2023). These sub-systems are directed by domain-general central executive resources.

The interactive use of working memory and long-term memory resources can boost working memory capacity (for a review, see Hart et al., 2024) by maximizing the resources available to encode, maintain, and/or retrieve information, rather than relying on domain-specific processing and storage within working memory (Brown & Wesley, 2013). Training people to use semantic strategies during working memory tasks may therefore be a promising approach to boosting capacity. However, we still need to investigate the effect of semantic strategy training in visual working memory (i.e., memory for visual representations such as patterns, colours, orientations, etc.). Furthermore, visual working memory is particularly vulnerable to aging (Johnson et al., 2010; Logie & Maylor, 2009). Interventions aimed at enhancing visual working memory capacity could potentially have a greater impact on older adults and help to reveal mechanisms underlying cognitive aging. Therefore, the present research was aimed at establishing young and older adults’ use of semantic strategies and the extent to which these age groups may benefit from semantic strategy instruction during visual working memory tasks.

### Strategic approach during working memory tasks

A ‘strategy’ is a procedure, or set of procedures, that can be used when performing cognitive tasks (Lemaire, 2016) and which likely impact capacity (e.g., Belletier et al., 2023; Bengson & Luck, 2016; Brown & Wesley, 2013; Gonthier & Thomassin, 2015; Laine et al., 2024; Morrison et al., 2016; Nicholls & English, 2020). Previous studies have therefore asked participants to self-report strategy use during visual working memory tasks (e.g., Brown & Wesley, 2013; Forsberg et al., 2020a; Gonthier & Roulin, 2020; Gonthier & Thomassin, 2015; Laine et al., 2018; Nicholls & English, 2020; Ozimič et al., 2023). In a recent review, Gonthier (2021) identified categories of strategies that participants can use during visuospatial working memory tasks, including: ‘chunking’ individual items together; visuospatial rehearsal; verbal recoding; and using semantics. Notably, these strategies are not necessarily mutually exclusive. A participant may rely on a single process (e.g., simple visuospatial rehearsal could be performed without any other strategy) but, more realistically, they may combine multiple strategies (Gonthier, 2021). Furthermore, there is significant overlap between some approaches. Strategies such as verbal recoding and using semantics, for example, can be expected to co-occur (e.g., Lewis-Peacock et al., 2015). Notably, though, these are dissociable approaches. An individual may verbally rehearse semantic labels. However, verbal rehearsal is not needed to benefit from meaningful or multimodal stimuli (Brady et al., 2016; Brady & Störmer, 2022; Brown & Wesley, 2013; Chung et al., 2023; Delogu et al., 2009; Plaska et al., 2021).

Strategies that are often associated with greater memory performance are those that involve actively manipulating the contents of working memory and creating associations to strengthen the memory trace (Laine et al., 2024; McNamara & Scott, 2001). This could include grouping information based on meaningful connections and using long-term memory resources (i.e., semantics; McNamara & Scott, 2001). An example of a semantic-based strategy reported during verbal working memory tasks is elaboration (e.g., Bailey et al., 2009, 2011; Bartsch & Oberauer, 2021; Dunlosky & Hertzog, 2001; Dunlosky & Kane, 2007). This involves enriching the memory trace by activating its meaning and linking it to deeper semantic associations (e.g., forming sentences, mental imagery), rather than focussing on the low-level stimulus features (Bartsch & Oberauer, 2021). Those who report engaging in such a strategy typically show improved performance (Bailey et al., 2011; Dunlosky & Kane, 2007). Recently, Ozimič et al. (2023) interviewed participants regarding their strategy use during a change detection task. Participants reported many strategies, one of which was ‘pattern recognition’, involving immediately becoming aware of a pattern within the stimuli. Particularly, participants most frequently reported this strategy during spatial position encoding. Qualitative analysis revealed that this strategy helps to encode stimuli together, with one participant noting that the perceived familiarity of the array made it easy to remember. This demonstrates the strategic involvement of semantic long-term memory during visuospatial working memory tasks. Importantly, though, there are individual differences in the availability of specialized cognitive resources relevant to using particular strategies, due to the role of central executive resources in strategy implementation (Gonthier & Roulin, 2020; Logie, 2011; Logie et al., 2021). Therefore, it could also be that participants who have higher spans have more capacity to successfully implement and benefit from semantic strategies (Bartsch & Oberauer, 2021).

However, despite the perceived usefulness, using semantic strategies may not be common during working memory tasks. Some studies have found that only around a quarter of participants spontaneously elaborate (Bailey et al., 2011; Bartsch & Oberauer, 2021; Dunlosky & Kane, 2007). In contrast, some people use a less efficient strategic approach or do not use strategies at all when completing working memory tasks. This is considerably less beneficial to their performance (Bailey et al., 2011; Dunlosky & Kane, 2007; Laine et al., 2024). Despite the experimental evidence showing the positive effect of mnemonics on working memory performance, our understanding of cognitive strategies remains limited (Lemaire, 2016; von Bastian et al., 2022). Furthermore, we need more evidence about strategy use during visual working memory tasks in particular.

### Visual matrix tasks and the benefit of visual semantics

**Fig. 1:**
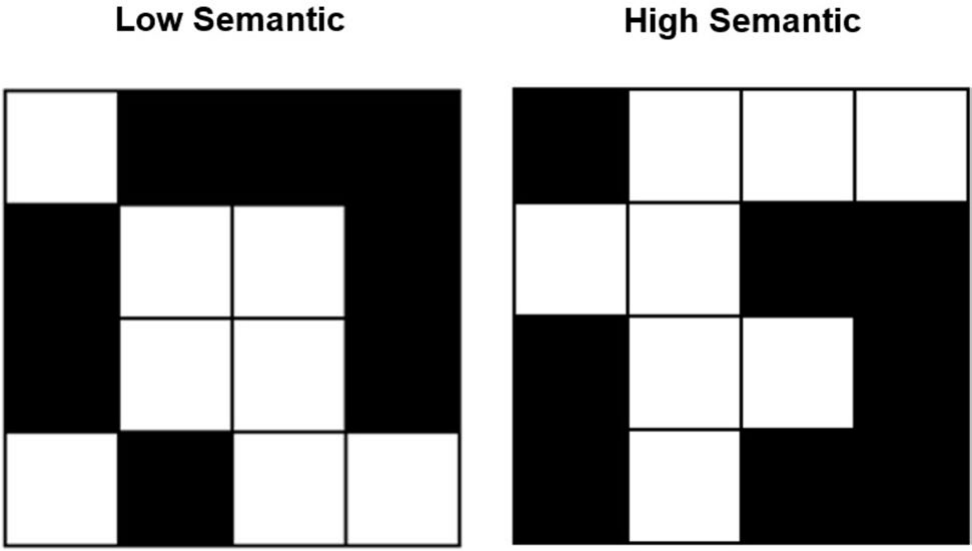
Example of ‘low semantic’ and ‘high semantic’ stimuli from the modified Visual Patterns Test (Brown et al., 2006) taken from task level of complexity eight (eight black cells for recall). For example, the high semantic pattern in this example may resemble an ‘i’, ‘back-to-front c’, or an ‘F’ (when considering both the black and white cells)

Visual ‘matrix’ tasks are commonly used to measure visual working memory capacity (e.g., Beigneux et al., 2007; Brown & Wesley, 2013; Nicholls & English, 2020; Phillips & Baddeley, 1971; Williamson et al., 2011). During the Visual Patterns Test (VPT; Della Sala et al., 1997, 1999), participants briefly view a series of black-and-white checkered patterns which gradually increase in size and complexity. After offset of a given stimulus and a short delay period, participants attempt to recall the pattern. Using a ‘span’ procedure, the task continues until participants are unable to reliably recall the patterns at a given level of complexity. Brown et al. (2006) created ‘low semantic’ and ‘high semantic’ versions of this task, based on participants’ ability to attach verbal labels to configurations within the patterns. The most abstract (low semantic) and the most meaningful/verbalizable (high semantic) patterns available at each level of complexity were selected for each new task version (see Fig. 1). For example, high semantic patterns may more frequently resemble letters or numbers, or even more elaborate configurations such as objects or animals, particularly as the patterns increase in size and complexity (Brown et al., 2006).

Research in the verbal domain has consistently found that using meaning within stimuli (i.e., semantics) can help to ‘free up’ (Kowialiewski et al., 2021) or supplement working memory resources. For example, meaningful sentences are easier to recall than abstract sentences, demonstrating semantic long-term memory support for verbal working memory (e.g., Meltzer et al., 2016). It is now established that pre-existing semantic representations can also support visuospatial working memory performance (for reviews, see Chung et al., 2024; Hart et al., 2024). Indeed, visual matrix patterns are typically better recalled when they offer more semantic availability (e.g., Brown & Wesley, 2013; Hamilton et al., 2018; Nicholls & English, 2020; Nicholls & Stewart, 2023; Orme et al., 2017; Riby & Orme, 2013). For example, Brown and Wesley found that enhanced recall (i.e., pattern reconstruction) associated with the high semantic/verbalizable patterns in the modified VPT (Brown et al., 2006) withstands articulatory suppression. This suggests that verbal rehearsal via the phonological loop is not the source of the performance benefit. Rather, the activated semantic representations are likely supporting the temporary storage of the abstract information in working memory (Brown & Wesley, 2013).

Brown and Wesley (2013) also showed that young adults who reported combining visual and verbal strategies during the modified VPT (Brown et al. 2006) exhibited increased capacity (see also Souza & Skóra, 2017). Though, perhaps counter-intuitively, the ‘combiners’ outperformed the ‘non-combiners’ on the low semantic, rather than the high semantic task. However, this aligns with theory. Semantic representations may be automatically activated upon perception of a meaningful stimulus (Logie, 2011; see also Forsberg et al., 2019; Orme et al., 2017; Plaska et al., 2021). It is also possible, though, to use executive resources strategically to ‘seek out’ meaning within more abstract visual representations and retrieve stored semantic knowledge (Brown & Wesley, 2013). Therefore, when there was higher semantic availability, specifically the non-combiners’ performance benefitted relative to their performance during the more abstract, low semantic task. In contrast, the combiners were able to perform well on both the low and the high semantic tasks. This was presumably due to using their efficient, active strategic approach to retrieve and associate stored semantic knowledge, boosting capacity, even for the more difficult, low semantic patterns.

Considering behavioural evidence, higher semantic availability in visual matrix tasks improves change detection accuracy and processing speed (Mammarella et al., 2014; Riby & Orme, 2013) and benefits recall, at least in young adults (Brown & Wesley, 2013; Hamilton et al., 2018; Nicholls & English, 2020). Neuroimaging (event-related potential (ERP)) evidence demonstrates that high semantic stimuli are associated with less early-stage/low-level visual processing and lower memory encoding load for young adults, due to the involvement of long-term knowledge and more easily ‘unitized’ visual configurations (Orme et al., 2017; Riby & Orme, 2013). In contrast, low semantic stimuli involve more complex, time-consuming retrieval processes, driven by executive resources and with more evidence of uncertainty and post-retrieval monitoring (Orme et al., 2017; Riby & Orme, 2013). However, high semantic stimuli also appear to require more active, later stage visual working memory processing resources, presumably to combine and maintain both the visual and the semantic content (see Bor et al., 2003). For example, studies using electroencephalography (EEG) have demonstrated increased delay activity during the retention period for meaningful versus abstract stimuli (Asp et al., 2021; Brady et al., 2016).

Executive resources are therefore likely to be involved in both low and high semantic visual working memory tasks, but in different ways. Indeed, Brown and Wesley (2013) found that administration of a central executive suppression task during maintenance (random tapping; e.g., Darling et al., 2007) removed the benefit of high semantic availability in the modified VPT (Brown et al., 2006). This shows that there is a cognitive cost to combining modalities (e.g., verbal, visual, semantic), due to the association and/or rehearsal of meaningful representations in the context of the specific, abstract visual pattern (Brown & Wesley, 2013; Riby & Orme, 2013; see also Brown et al., 2012). Although executively demanding, this approach may help to reduce the memory load associated with more difficult, abstract configurations and reduce the resources required for both early-stage and retrieval processes. Importantly, though, we require more evidence regarding the extent to which people spontaneously use semantic strategies when performing visual working memory tasks, as existing research is limited (Gonthier, 2021). Previous research gathering data on spontaneous strategies in the visual domain has often focused on visual versus verbal approaches (e.g., Brown & Wesley, 2013; Nicholls & English, 2020).

### Semantic strategy training in working memory

Manipulation of strategic approach during working memory tasks is required to identify whether active use of specific strategies could be beneficial for performance. As such, previous studies have attempted to ‘train’ participants to use specific strategies, showing some promise for potentially boosting working memory task capacity (e.g., Allen et al., 2021; Atkinson et al., 2018; Bengson & Luck, 2016; Forsberg et al., 2020b; Laine et al. 2018; Souza & Skóra, 2017). However, any benefits do not typically ‘transfer’ to untrained or more generalised working memory tasks (e.g., Forsberg et al., 2020a).

The use of specifically semantic strategies during working memory tasks could improve participants’ ability to detect and construct meaningful patterns within abstract stimuli, facilitating retrieval (Richter et al., 2015). However, evidence regarding the effect of semantic strategy training on capacity is mixed (Bailey et al., 2009; Bartsch et al., 2019; Bartsch & Oberauer, 2019, 2021; Campoy & Baddeley, 2008; McNamara & Scott, 2001; Miotto et al., 2013, 2020; Turley-Ames & Whitfield, 2003). For example, in verbal working memory, instructing participants to strategically organize random words into meaningful categories can improve memory for word lists (Miotto et al., 2013, 2020). Yet, other studies show that instructing elaborative strategies, such as mental imagery and sentence generation, do not benefit performance (Bailey et al., 2009; Bartsch et al., 2019; Bartsch & Oberauer, 2019, 2021; McNamara & Scott, 2001; Turley-Ames & Whitfield, 2003). It may be that certain methods of elaboration (e.g., sentence generation) require the processing of additional irrelevant material, which could hinder recall of task-relevant words (Bartsch & Oberauer, 2021), particularly for those with lower pre-training capacity (Turley-Ames & Whitfield, 2003).

To our knowledge, the effects of semantic strategy training in the visuospatial domain have not yet been investigated. Strategic approach and training clearly show good potential to benefit visual working memory capacity, but more comprehensive research is needed. For example, due to individual differences in executive resources and capacity, strategy instructions may not be implementable by all participants. The challenge therefore remains to establish reliable, effective strategy training protocols, and avoid the possibility of reduced performance in some cases (e.g., Nyberg et al., 2003).

### Summary

Previous research has demonstrated a facilitative effect of high semantic availability in visual matrix tasks assessing both pattern recognition (e.g., Riby & Orme, 2013; Mammarella et al., 2014) and recall (e.g., Brown & Wesley, 2013; Nicholls & English, 2020). One of the most important questions related to this effect is whether we can instruct participants to implement and benefit from semantic strategies. Semantic strategy training has previously been successful in boosting verbal working memory (e.g., Miotto et al., 2013, 2020), but requires greater understanding, particularly in the visuospatial domain.

Across two experiments, this research aimed to contribute to the existing literature by investigating whether instructing participants to use a semantic strategy during a visual working memory task can benefit task performance. The findings will inform whether semantic strategy training could be a promising intervention for boosting visuospatial working memory capacity. Importantly, we assessed whether this could apply to young adults and/or healthy older adults with lower initial capacity and resources. We also explored both young and older adults’ spontaneous strategy use during visual matrix task performance, including specifically semantic strategy use.

## Experiment 1

Experiment 1 was a pilot study aimed at investigating the effects of semantic availability and a novel semantic strategy instruction protocol in young adults. Specifically, participants were encouraged to seek out and use meaning within the stimuli to try to maximize their task performance. We predicted effects of semantic availability and semantic strategy instruction on capacity. Furthermore, we predicted an interaction in that the benefit of strategy instruction would be greatest in the more challenging, low semantic task version (Brown & Wesley, 2013). This is because, in that task version, there should be less automatic activation of semantics and instruction should increase the extent to which participants actively seek out meaningful representations. In other words, the instructed group were expected to perform relatively well across both low and high semantic tasks using the instructed strategic approach, whereas the control group were expected to exhibit lower capacity in the more difficult, low semantic task, in which semantic codes are less automatically activated (Brown & Wesley, 2013).

### Methods

#### Participants

The study was ethically approved by the Department of Psychological Sciences and Health Ethics Committee at the University of Strathclyde. A power analysis was carried out using G*Power (Faul et al., 2009), driven by paired *t*-tests for investigating effects of semantic availability within each instruction group. A total sample of 44 gives .90 power (1 – β) to detect a medium effect size (*dz* = .5; *α* = .05; two-tailed).^1^ The total final sample comprised 44 participants. Note, three participants were excluded and replaced due to being identified as extreme outliers via boxplots (i.e., scoring above or below the upper or lower quartile, plus 3 × the interquartile range). The participants were aged 18–35 years (*M* = 24.68, *SD* = 3.66) with a mean number of years of education of 16.52 (*SD* = 1.98). Within the sample, 12 reported identifying as male (27.3%), 31 as female (70.5%), and one responded *other/prefer not to say*. Participants self-reported being based in the UK, having no memory or uncorrected vision impairments, and having access to a compatible computer (desktop or laptop) to participate in the study remotely.

#### Design

A 2 × 2 mixed factorial design was used to investigate the effects of semantic strategy instruction (control or instructed; between groups) and semantic availability (low or high; repeated measures) on visual working memory recognition accuracy (proportion correct) and response time (RT; ms).

#### Materials

##### Modified Visual Patterns Test (VPT)

Modified versions of the VPT (Della Sala et al., 1997), with low or high availability of visual semantics, were used (Brown et al., 2006). Stimuli were black-and-white checkered patterns consisting of half black and half white cells on a matrix grid. Matrix size increased across task levels, beginning at level four (four black cells to remember) through to level 15, with each level consisting of three trials. Shapes within the high semantic stimuli were more likely to have been reported as resembling meaningful items such as letters, numbers, objects, or animals (see Fig. 1; Brown et al., 2006).

The task was computerized and, due to COVID-19 restrictions, administered remotely using E-Prime Go (Psychology Software Tools, Inc., 2020). Performance was measured by recognition accuracy (e.g., Riby & Orme, 2013). Test items for lure trials were created via three methods: (1) selecting a random black cell and moving it to the closest available white grid space; (2) adding an additional black cell into a randomly selected white grid space; or (3) reverting a randomly selected black cell to a white cell.^2^

##### Strategy instructions

Participants were given standard instructions, including the same two standard, example stimuli (level four; Della Sala et al., 1997; Brown et al., 2006). All participants were informed that, when approaching the task, it is possible to use a variety of strategies and that they would be asked to report these at the end of the session. The instructed group were additionally made aware of the possibility of using a semantic strategy and its expected effectiveness (i.e., “One way to support performance of this task is to activate and use any meaning or familiarity contained within the patterns, such as letters, symbols, or even everyday objects or animals”). These participants were then briefly instructed on how to use this strategy (i.e., “For example, in the pattern on the left above, you may notice that the black cells resemble a letter ‘T’ on its side… You can try to use that knowledge to help you remember what the patterns looks like. Even if you don’t notice anything meaningful relatively automatically or straight away, you could try to search for meaning”; see Online Supplementary Material (OSM) for the task instructions by instruction condition). The instructed participants were asked to use a semantic strategy as much as possible, and to do so alongside any other strategies they may find useful. This was specifically to avoid removal of other useful strategies, such as visual rehearsal, and with the aim of avoiding negative impacts on performance. No participants were informed of any distinction between the two task versions they were completing, only that they would be asked to carry out a second task which “will essentially be the same as the first”.

##### Strategy use questionnaire

A Likert-style questionnaire was administered to record self-reported strategy use upon completion of the tasks. Participants reported the extent to which they relied on various strategies (i.e., verbal, visual, and semantics). The first question used a five-point continuum for participants to rate the extent to which they used a verbal and/or visual strategy (1 = *verbal only*, 5 = *visual only*). The remaining seven questions used a five-point Likert scale (1 = *never*, 5 = *always*) and addressed the extent to which participants: “combined verbal and visual strategies”; “‘counted up’ black or white cells”; “used verbal labels to rehearse a pattern” (verbal recoding); “noticed meaningful or familiar shapes within a pattern” (automatic semantic activation); “actively tried to find meaningful or familiar shapes within a pattern” (active use of semantics); “used meaningful or familiar information to remember a pattern” (overall semantic strategy use); and “refreshed their mental image of the pattern” (visual refreshing; see OSM for the full semantic strategy questionnaire). Note, the three semantic strategy questions are unique additions to the questionnaire devised by Brown and Wesley (2013; see also Nicholls & English, 2020).^3^

#### Procedure

Participants were assigned, via a randomly generated number list using Microsoft Excel, to either the control or semantic strategy instruction condition. Each participant was administered both the high and low semantic versions of the modified VPT (Brown et al., 2006). Task order was fully counterbalanced across the sample.

Participants began the tasks at level four (the same level as the example patterns; Brown & Wesley, 2013; Della Sala et al., 1997). Due to the recognition nature of the task, trials continued through to the maximum level 15. Participants took part in their own chosen location, usually their home, and accessed the task programmes via a link provided by the researcher. They were asked to minimize potential for distractions, close off any other computer programmes, and display their screen on one active monitor before beginning. Participants began a trial by pressing the spacebar, then a fixation cross would appear for 2 s. This was then replaced by the stimulus upon a white background for 1,500 ms (Riby & Orme, 2013), followed by a 10 s blank (white) screen (Brown et al., 2006; Della Sala et al., 1997). After the delay period, a test pattern was displayed and participants were asked to indicate whether the probe was the same as or different to the original pattern (by pressing the ‘M’ or ‘Z’ key on their keyboard, respectively; see Fig. 2). When one task version had finished, participants were offered a short break before beginning the next version, which was performed in the same way. Finally, participants completed the strategy questionnaire. They were then thanked and debriefed.

**Fig. 2:**

Example trial from Experiment 1, in which the correct response was ‘same’ (i.e., the test item had been presented earlier in the trial). Participants initiated each trial by pressing the spacebar, after which a fixation cross and then the to-be-remembered pattern was presented, followed by a retention period (blank screen). Participants then responded to the test probe as ‘same’ or ‘different’, via key-press. *Note.* Stimuli are not drawn to scale

#### Data analyses

For both experiments presently reported, data were primarily analysed using SPSS version 28. JASP 0.18.3 (JASP Team, 2024) was additionally used to determine Bayes factors, which are provided as a supplement to the frequentist analyses. *BF*_incl_ indicates the strength of the evidence for including each factor or interaction in the model. *BF* < 1 indicates support for the null hypothesis. *BF* = 1–3 is considered as indicating weak or anecdotal evidence, *BF* = 3–10 as substantial evidence, and *BF* > 10 as strong evidence (Wetzels et al., 2011).

#### Transparency and openness

We report how we determined our sample sizes and any data exclusions for both of the present experiments. All data and analysis syntax are openly available at the Open Science Framework project ‘Semantic availability and strategy training in visual working memory’ (https://osf.io/aemb7/). The stimuli from the modified VPT (Brown et al., 2006) are also openly available on the Open Science Framework (https://osf.io/fg3rc/). The strategy instructions and strategy questionnaire can be accessed in the OSM. The study design, hypotheses, and analysis plans for both Experiment 1 (10.17605/OSF.IO/YJRCZ) and Experiment 2 (10.17605/OSF.IO/MXDZV) were pre-registered prior to data collection.

### Results

The mean accuracy (proportion correct) and RT data across conditions are presented in Table 1. These are mostly identical across groups but accuracy is numerically higher in the instructed group for the high semantic task.

**Table 1:** Mean accuracy scores (proportion correct) and response times (ms; both with SDs) for the control and instructed groups across low and high semantic tasks in Experiment 1

		Low semantic	High semantic
Accuracy	Control	.82 (.08)	.82 (.09)
	Instructed	.82 (.10)	.85 (.09)
Response time, ms	Control	2,266 (923)	2,444 (1,105)
	Instructed	2,290 (991)	2,334 (1,140)

A 2 (task semantic availability; low, high) × 2 (strategy instruction; control, instructed) mixed analysis of variance (ANOVA) revealed no main effect of semantic availability on accuracy, *F*(1,42) = 1.81, *MSE* = .004, *p* = .185, η^2^_p_ = .041, *BF*_incl_ = .47, with no reliable difference between the low (*M* = .82; *SD* = .09) and high (*M* = .84;* SD* = .09) semantic pattern sets. There was also no main effect of semantic strategy instruction on accuracy, *F*(1,42) = 0.18, *MSE* = .013, *p* = .676, η^2^_p_ = .004, *BF*_incl_ = .38, with the control (*M* = .82; *SD* = .02) and instructed (*M* = .83, *SD* = .02) groups performing similarly overall. Importantly, there was no significant interaction between semantic availability and semantic strategy instruction, *F*(1,42) = 1.17, *MSE* = .004, *p* = .286 , η^2^_p_ = .027, *BF*_incl_ = .44. The same mixed ANOVA on the RT data revealed no significant effects (all *p* > .43, all *F* < .63, all *BF*_incl_ < .38).

#### Exploratory analyses

##### Accuracy and RT data

An exploratory analysis considering the effect of pattern size (Brown et al., 2006) revealed no significant effects on performance beyond the expected main effect of pattern size (i.e., negative effect of increased pattern size on accuracy; see OSM). Similarly, when including pattern size in the analysis with RT as the outcome variable, only the main effect of pattern size emerged (see OSM). Another exploratory analysis considering the effect of administration order (Nicholls & English, 2020) revealed no significant effects or interactions involving this variable (see OSM).

##### Strategy use

Strategy data are displayed in Table 2. Notably, strategy reports were similar across groups. Importantly, regarding semantics, both the control and instructed groups reported automatically noticing and actively searching for meaningful shapes at least sometimes. Furthermore, both groups reported overall use of visual semantics, regardless of how they were initially activated.

**Table 2:** Participants’ median response values (presented along with their numerical value and the interquartile range) to strategy questions in Experiment 1, by semantic strategy instruction condition

	Control	Instructed
Overall strategy	Mostly visual (4, 2.25)	Equally verbal and visual (3, 2)
Combining	Sometimes (3, 2)	Sometimes-mostly (3.5, 1)
Counting up	Rarely (2, 2)	Sometimes (3, 2)
Labelling	Mostly (4, 1.25)	Sometimes-mostly (3.5, 1)
Automatic semantics	Sometimes-mostly (3.5, 1)	Sometimes (3, 1.25)
Active semantics	Sometimes (3, 1.25)	Mostly (4, 2)
Overall semantics	Mostly (4, 1.25)	Mostly (4, 1.25)
Visual refreshing	Mostly (4, .50)	Mostly (4, 2)

*Note.* For Q1 (overall strategy), 1 = *verbal strategy only*, 5 = *visual strategy only*; for all other Qs, 1 = *never*, 5 = *always*

Mann-Whitney *U* tests were used to test for any reliable differences in strategy use between the control and instructed group. These were only significantly different for ‘counting up’, *U* = 147.50, *z* = −2.29, *p* = .022 (mean rank, control = 18.20; mean rank, instructed = 26.80; all other *U* ≥ 193.50, all other *p* > .22).

Spearman’s correlations were also used to estimate relationships between strategy reports and task performance for both groups (see OSM). However, due to the very limited sample size, these correlations will not be sufficiently stabilized to determine meaningful relationships (Schönbrodt & Perugini, 2013).

### Discussion

Experiment 1 provided an initial investigation of the effect of visual semantics and semantic strategy instruction on young adults’ visual working memory performance. The key findings are that there were no experimental effects of semantic availability or semantic strategy instruction on capacity. Furthermore, semantic strategy instruction neither boosted, nor hindered, task accuracy.

Numerically, the instructed groups’ performance was higher in the high semantic task, but we did not observe a reliable benefit of semantic availability. This was unexpected, as previous studies have consistently observed boosts to young adults’ capacity in high semantic versus low semantic matrix patterns (e.g., Brown et al., 2006; Hamilton et al., 2018; Nicholls & English, 2020; Riby & Orme, 2013). However, it is worth noting that the majority of these studies have measured recall instead of recognition (e.g., Brown et al., 2006; Brown & Wesley, 2013; Della Sala et al., 1997, 1999; Hamilton et al., 2018; Nicholls & English, 2020). Similarly, in verbal working memory, there is a sentence superiority effect on word list recall that is smaller and less consistent during recognition (Allen et al., 2018). Indeed, the retrieval processes involved during working memory tasks are different for these paradigms. During recognition tasks, participants make familiarity-based judgments. This may automatically re-activate stored representations, relying less on central executive resources than recall, and/or being generally less sensitive to differences associated with semantic properties of the stimuli. During recall tasks, participants reconstruct stimuli based on the memory trace, which is more challenging and may benefit more from greater semantic availability. It is also possible that the associated shorter encoding time in this recognition task limited the potential for semantic coding.

Additionally, as per earlier research, there were individual differences in strategy use (Brown & Wesley, 2013; Nicholls & English, 2020; for a review, see Gonthier, 2021). Uniquely, the present study additionally shows that young adults generally report using a semantic strategy at least sometimes, to the same extent as they report using visual refreshing, for example. Furthermore, considering semantic strategy instruction, strategy reports between the control and instructed groups were similar. Only the ‘counting up’ strategy exhibited a significant difference across the groups, with the instructed group reporting greater use of this strategy. This was unexpected, as counting up has been previously regarded as an inefficient strategy that should not in itself benefit memory for the visual array (Nicholls & English, 2020). However, this may be a useful approach when considering pattern recognition, particularly when many of the lure stimuli contain a different number of cells as the original pattern. This is unlikely, though, particularly at larger pattern sizes, given the brief encoding time available. It may be that the instructed group combined the semantic strategy with this strategy, by counting up the number of cells making up a meaningful shape. For example, participants may have identified that a collection of cells in the matrix grid resembled the letter ‘L’ and retained the number of horizontal and/or vertical cells within this configuration. Notably, though, the instructed group did not report using a counting up strategy extensively (i.e., only *sometimes* vs *rarely* in the control participants). The instructed group may have also reverted to a counting up strategy if they were finding the instructed semantic strategy too difficult to implement. Finally, it is important to highlight that, not only was the instruction protocol not associated with any deficits in task performance, but there were also no other discernible negative effects on reported strategies.

Next, it was important to carry out a laboratory-based study to investigate the effects of semantic availability and semantic strategy instruction on visual matrix task performance using a recall paradigm. We also wanted to investigate the extent to which the impacts of strategy instruction may differ when considering performance of participants with lower initial task capacity (in this case, older adults).

## Experiment 2

Visual working memory is highly age-sensitive (Johnson et al., 2010; Logie & Maylor, 2009). Indeed, in visual matrix tasks, performance reliably declines with age (Beigneux et al., 2007; Nicholls & English, 2020), showing the steepest change amongst a variety of other ‘fluid’ cognitive abilities (Johnson et al., 2010; Logie & Maylor, 2009). Furthermore, older adults do not benefit from the availability of semantics in visual matrix tasks as reliably as young adults (Hamilton et al., 2018; Nicholls & English, 2020).

Nicholls and English (2020) administered low and high semantic task versions of the modified VPT (Brown et al., 2006) with young and older adults. Although both age groups more accurately recalled the high semantic patterns, older adults did not differentially benefit from high semantic availability, despite having lower initial capacity (see also Hamilton et al., 2018, who showed less benefit for older adults). As previously discussed, benefitting from high semantic availability appears to involve central executive resources (Brown & Wesley, 2013). Therefore, the availability and efficacy of limited attentional resources in older age could potentially account for this. Nicholls and English also showed that older adults reported relying primarily on visual refreshing and, beyond this, used less efficient alternatives. Specifically, older adults also reported relying on counting up the cells in matrix patterns, which could potentially hinder capacity for visual details, especially for more complex patterns (see also Forsberg et al., 2020a). This was in comparison to young adults, who reported a flexible and more efficient strategic approach including multimodal coding (i.e., combining strategies). Therefore, inefficiencies in strategic approach in older age could at least partly account for aging effects on capacity (Naveh-Benjamin & Cowan, 2023).

Strategy training in older age seems possible. The Scaffolding Theory of Aging and Cognition posits that older adults have the potential to use more generalized attentional resources to compensate for specialized cognitive resources that are more vulnerable to decline. That is, generalized resources can ‘scaffold’ specialized cognitive resources such as visual storage and processing (Park & Reuter-Lorenz, 2009; Reuter-Lorenz & Park, 2010). Furthermore, attentional resources could facilitate strategy development and execution (Logie, 2011). Following strategy training of a visuospatial strategy for verbal memory, Nyberg et al. (2003) showed that young adults exhibited boosted capacity, whereas older adults showed no benefit. However, when older adults were grouped depending on their level of neural engagement related to the relevant strategy, those who exhibited this functional ability showed marked improvements to capacity after training. In contrast, older adults whose neuroimaging profiles suggested an inability or failure to engage with the trained strategy exhibited decreased capacity. Allen et al. (2021) also showed that, after training, both young and older adults were able to direct attention to items deemed more valuable during a visual working memory task. Despite older adults having poorer memory overall, they showed no greater benefit compared to young adults, but their performance was shifted further away from floor levels.

The present Experiment 2 aimed to establish whether older adults could differentially benefit from semantic availability and semantic strategy instruction. This is important as existing evidence on the effect of semantic availability on older adults’ visual working memory task performance is mixed. Furthermore, semantic strategy instruction in visuospatial working memory, not yet investigated in older adults, could encourage them to encode stimuli in a more holistic and meaningful way, creating a more robust representation in memory. We also explored young and older adults’ spontaneous strategy use, and particularly semantic strategies, which requires direct investigation. We predicted that older adults would exhibit decreased memory capacity compared to young adults. Furthermore, we expected high semantic availability and semantic strategy instruction to boost capacity. Importantly, we also predicted a three-way interaction, with the benefit of semantic strategy instruction being greatest for older adults in the low semantic task. This is because there should be less automatic activation of semantics in this more difficult task version. Strategy instruction should allow participants to seek out semantics more actively in this case, boosting capacity, especially for older adults who have lower initial capacity and more potential benefit to gain.

### Methods

#### Participants

The study was approved by the Department of Psychological Sciences and Health Ethics Committee at the University of Strathclyde. A power analysis carried out using G*Power (Faul et al., 2009) indicated a required sample size of 128 participants to observe a main effect or interaction with a medium effect size (*f* = .25; *α* = .05) with .80 power (1 – β). The final sample comprised 128 participants. Note, five participants were replaced due to being outliers on at least one of the measures as determined by boxplots (i.e., scoring above or below the upper or lower quartile, plus 3 × the interquartile range), or due to administration error.

Participants self-reported being either 18–35 or 60+ years, not diagnosed with cognitive impairments or neurological conditions, and fluent in English (see Table 3 for demographics). Young adults were primarily undergraduate students recruited through the University participant pool who received course credits, and through social media advertising and word-of-mouth. Older participants were recruited through local participant panels and word-of-mouth. They volunteered on the basis of being cognitively healthy and received no incentives for participation. Older adults were screened for cognitive impairment using the Mini-Cog (Borson et al., 2000), with no participants exhibiting signs of impairment. The difference in years of education between age groups was significant, *t*(126) = −2.19, *p* = .030, *BF*_10_ = 1.64, being higher in the older adults, although note that the Bayes factor provides only weak evidence for this effect. Furthermore, estimated full-scale IQ was significantly higher in older adults, *t*(126) = −10.81, *p* < .001, *BF*_10_ = 3.08 × 10^16^. This can often be observed using verbal-based IQ estimates, due to increased verbal knowledge with age. Importantly, these differences are in the opposite direction of any expected age effects on memory.

**Table 3:** Demographic data of the participant samples from Experiment 2

		Young (18–34 y)	Older (60–87 y)	Overall
Age, y (*M* ± *SD*)		22.00 (3.98)	70.88 (6.72)	46.44 (25.14)
Gender (N, % of sample)	Male	13 (20.3%)	14 (21.9%)	27 (21.1%)
	Female	51 (79.7%)	49 (76.6%)	100 (78.1%)
	Other (non-binary)	0 (0%)	0 (0%)	0 (0%)
	Prefer not to say	0 (0%)	1 (1.6%)	1 (0.8%)
Education, y (*M* ± *SD*)		15.20 (2.41)	16.34 (3.39)	15.77 (2.99)
NART-Estimated IQ (*M* ± *SD*)		101.58 (6.52)	116.16 (8.60)	108.87 (10.55)

#### Design

The study took the form of a 2 × 2 × 2 mixed factorial design to investigate the effects of age (young, older), semantic strategy instruction (control, instructed; between participants), and semantic availability (low, high; repeated measures) on visual working memory capacity. The dependent variable was ‘mean span’, a more sensitive measure than the maximum span achieved. In both task versions, mean span was calculated by taking the mean size of the last three correctly recalled patterns for each participant (e.g., Brown et al., 2006; Della Sala et al., 1999; Nicholls & English, 2020). Where three correctly recalled patterns were not available, the mean was taken of the available successful trials.

#### Materials

##### Mini-Cog

The Mini-Cog (Borson et al., 2000) is a validated, brief cognitive functioning assessment for screening older adults for signs of unhealthy cognitive impairment. It involves assessing delayed verbal recall (three words) and clock-drawing ability (draw a clock face depicting a specified time).

##### National Adult Reading Test (NART)

The National Adult Reading Test (NART; Nelson & Willison, 1991) is a brief assessment to provide estimated full-scale IQ. This involves participants reading aloud a list of 50 words, which gradually become more difficult to pronounce. Participants were asked to try their best to attempt to pronounce all of the words, even if they were unsure.

##### Modified Visual Patterns Test

Two modified versions of the VPT (Della Sala et al., 1997) were used, one with high availability of visual semantics and one with low availability (Brown et al., 2006), as in Experiment 1. However, memory was assessed using recall. The task was computerized and administered using E-Prime 3.0 (Psychology Software Tools, Inc., 2016) and paper templates were used to facilitate recall. As in Experiment 1, participants were given standard instructions, including the same two example stimuli from level four (Brown et al., 2006; Della Sala et al., 1997; see OSM).

##### Strategy Use Questionnaire

The Likert-style strategy questionnaire from Experiment 1 was administered, this time via paper, to measure participants’ self-reported use of various relevant strategies (see OSM).

#### Procedure

Older adults first completed the Mini-Cog (Borson et al., 2000), which took approximately 5 min. All participants completed the NART (Nelson & Willison, 1991), which also took approximately 5 min. Participants were randomly assigned via a randomly generated list in Microsoft Excel to the control or instructed condition. All participants completed both low and high semantic versions of the modified VPT (Brown et al., 2006). Task order was fully counterbalanced across each age group and condition.

As in Experiment 1, within the instructions, participants were informed that at the end of the task they would be asked to report the extent to which they felt they used different strategies. Again, the instructed group were additionally informed about the possibility of a semantic strategy and its expected usefulness. They were also briefly asked to use this strategy alongside any other strategies they found useful (see OSM).

**Fig. 3:**
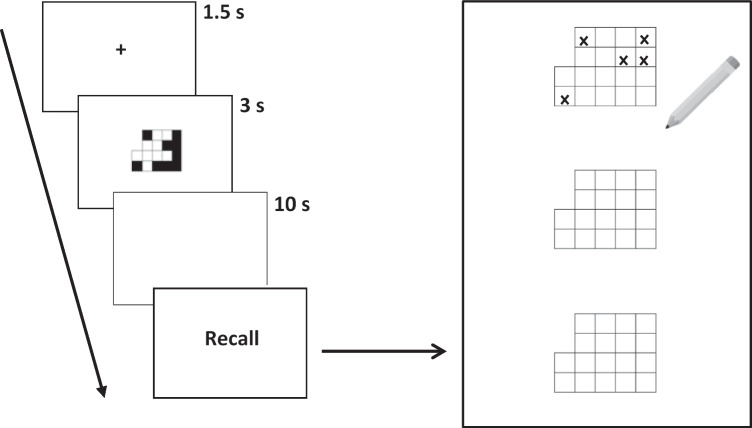
Example trial from Experiment 2. After a brief fixation cross, participants were shown a pattern for 3 s. After a 10 s delay, they were asked to attempt to recall the pattern using a blank paper template, by crossing out the cells that they remembered as having been black. *Note.* Stimuli are not drawn to scale. Recall templates were printed on standard A4 paper

After receiving task instructions and completing three standard practice trials from level four of the task (Brown et al., 2006; Della Sala et al., 1997; Nicholls & English, 2020), the first task version was carried out. Young and older adults began the task at level four and level two, respectively, reflecting differences in capacity and task duration/practice, which would have been emphasised more greatly if all participants commenced at level two (Beigneux et al., 2007; Brown et al., 2006; Logie & Maylor, 2009; Nicholls & English, 2020). In each trial, a fixation cross appeared on the computer screen followed by a visual matrix pattern for 3 s. Then, participants viewed a blank (white) screen for 10 s until the word ‘recall’ appeared. Participants then attempted to recall the pattern on a blank paper template by crossing out the cells in the matrix that they remembered to have been black (see Fig. 3). Participants continued through the task until they were unable to recall any of the three patterns in a level. When one task version had finished, participants were offered a short break before beginning the next version, which was performed in the same way, including the practice trials, but with the different pattern set. Finally, participants completed the strategy questionnaire. They were then thanked and debriefed.

### Results

**Fig. 4:**
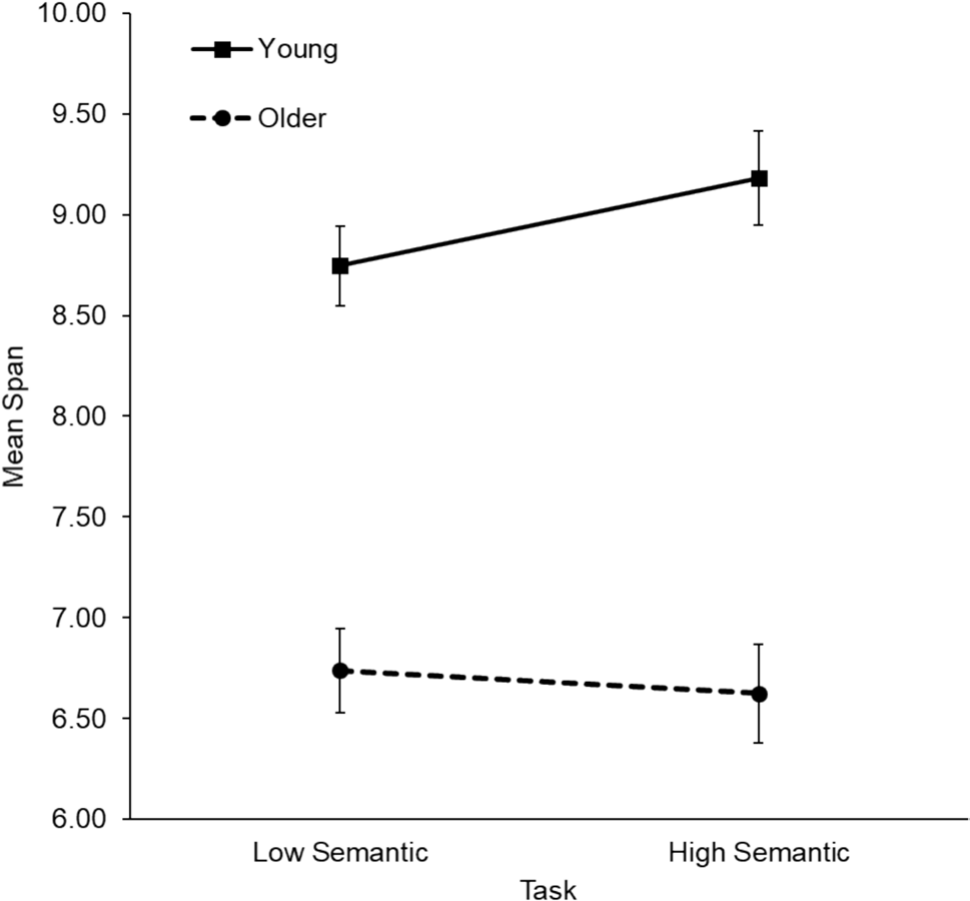
Mean span as a function of age group and semantic availability in Experiment 2 (with SEs)

Accuracy (mean span) data by participant group are displayed in Table 4. A 2 (age group: young, older) × 2 (semantic availability: low, high) × 2 (semantic strategy instruction: control, instructed) mixed-factorial ANOVA revealed a significant effect of age, *F*(1,124) = 62.10, *MSE* = 5.367 *p* < .001, η^2^_p_ = .334, *BF*_incl_ = 5.57 × 10^9^, with young adults (*M* = 8.96, *SD* = 1.56) having higher capacity than older adults (*M* = 6.68, *SD* = 1.69). There was no significant effect of semantic availability, *F*(1,124) = 1.61, *MSE* = 1.038, *p* = .207, η^2^_p_ = .013, *BF*_incl_ = .27, with similar performance across low semantic (*M* = 7.74, *SD* = 1.90) and high semantic (*M* = 7.90, *SD* = 2.31) tasks. There was also no significant main effect of strategy instruction, *F*(1,124) = 0.14, *MSE* = 5.367, *p* = .707, η^2^_p_ = .001, *BF*_incl_ = .31, with the control group (*M* = 7.77, *SD* = 1.99) and the instructed group (*M* = 7.88, *SD* = 1.99) having similar capacity. There was a significant two-way interaction between age and semantic availability, *F*(1,124) = 4.67, *MSE* = 1.038, *p* = .033, η^2^_p_ = .036, *BF*_incl_ = 1.48 (see Fig. 4), although note that the Bayes factor provided only weak evidence for this effect. The other two-way interactions (all *F* < 0.82, all *p* > .36, all *BF*_incl_ < .40) and the three-way interaction (*F* = 0.32, *p* = .570, *BF*_incl_ = .28) were not significant.

**Table 4:** Young and older adults’ mean capacity (span) scores (± SDs) from Experiment 2, for low and high semantic tasks by instruction group

		Low semantic	High semantic
Young adults	Control	8.77 (1.45)	9.17 (1.77)
	Instructed	8.72 (1.74)	9.20 (2.00)
Older adults	Control	6.72 (1.51)	6.42 (2.13)
	Instructed	6.76 (1.81)	6.83 (1.80)

*Note.* The maximum span that could be achieved was 15

To follow up the significant two-way interaction, planned comparisons (paired *t*-tests) assessed the effect of semantic availability within each age group. There was a significant effect of semantic availability in young adults, *t*(63) = −2.28, *p* = .026, *BF*_10_ = 1.50 (*M*_low_ = 8.75, *SD* = 1.59; *M*_high_ = 9.18, *SD* = 1.88) but not in older adults, *t*(63) = 0.69, *p* = .496, *BF*_10_ = .17 (*M*_low_ = 6.74, *SD* = 1.66; *M*_high_ = 6.62, *SD* = 1.97).

#### Exploratory analyses

##### Accuracy data

**Fig. 5:**
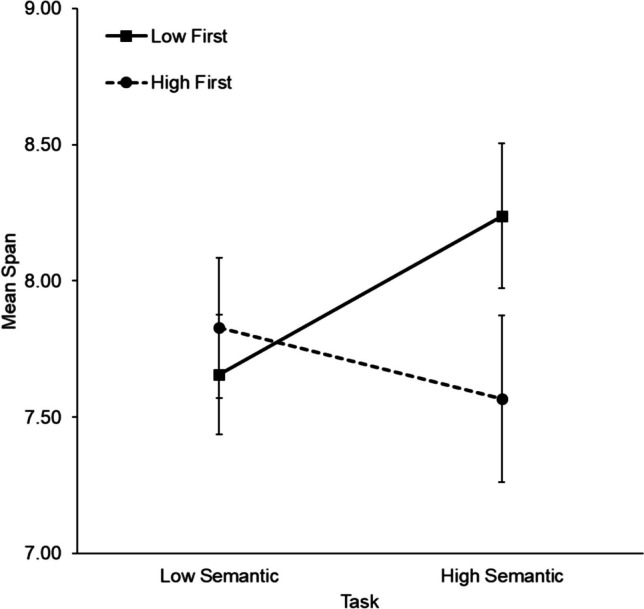
Mean span as a function of administration order and semantic availability in Experiment 2 (with SEs)

An exploratory 2 (age group: young, older) × 2 (semantic strategy instruction: control, instructed) × 2 (administration order: low semantic first, high semantic first) × 2 (semantic availability: low, high) mixed-factorial ANOVA was run to test the potential effect of administration order (Nicholls & English, 2020; data presented in the OSM). There was a significant interaction between semantic availability and administration order, *F*(1,120) = 11.74, *MSE* = .969, *p* < .001, η^2^_p_ = .089, *BF*_incl_ = 38 (see Fig. 5). There were no other significant effects or interactions involving administration order (all *F* ≤ 1.23, all *p* ≥ .270, all *BF*_incl_ < .57). Otherwise, the pattern of findings was the same as above, including the significant age × semantic availability interaction (*F* = 5.01, *p* = .027, *BF*_incl_ = 2.06).

To follow up the semantic availability × administration order interaction, Bonferroni-corrected paired *t*-tests were carried out to assess the effect of semantic availability within each administration order group (to meet significance, *p* < .025). There was a significant difference in capacity across the two tasks for those who completed the low semantic task first, *t*(63) = −3.53, *p* < .001, *BF*_10_ = 32 (*M*_low_ = 7.66, *SD* = 1.75; *M*_high_ = 8.24, *SD* = 2.13), but not for those who completed the high semantic task first, *t*(63) = 1.41, *p* = .163, *BF*_10_ = .35 (*M*_low_ = 7.83, *SD* = 2.05; *M*_high_ = 7.57, *SD* = 2.44).

##### Strategy use

Exploratory analyses investigated reported strategy use associated with age group and semantic strategy instruction. Table 5 illustrates that, in control participants, both young and older adults appear to rely on visual refreshing, but older adults tend to supplement this with other strategies including verbal labelling and use of semantics to a lesser extent than young adults. Considering the instructed group, strategy use appears to be more consistent across age groups, with older adults reporting more overall use of a semantic strategy than under control conditions. Yet, young adults still report more use of labelling and actively searching for meaningful configurations.

**Table 5:** Participants’ median response values (presented along with their numerical value and the interquartile range) to strategy questions in Experiment 2, by age group and semantic strategy instruction condition

	Young adults		Older adults	
	Control	Instructed	Control	Instructed
Overall strategy	Equally verbal & visual (3, 1)	Mostly visual (4, 1)	Mostly visual (4, 1.75)	Mostly visual (4, 1)
Combining	Sometimes-mostly (3.5, 2)	Sometimes (3, 1)	Mostly (4, 2)	Sometimes-mostly (3.5, 1)
Counting up	Sometimes (3, 1)	Sometimes (3, 2)	Sometimes (3, 2.5)	Sometimes (3, 2)
Labelling	Mostly (4, 2)	Mostly (4, 1)	Sometimes (3, 2)	Sometimes (3, 1)
Automatic semantics	Mostly (4, 2)	Sometimes (3, 2)	Sometimes (3, 1)	Sometimes (3, 1)
Active semantics	Sometimes (3, 3)	Mostly (4, 1)	Rarely-sometimes (2.5, 3)	Sometimes (3, 1.75)
Overall semantics	Sometimes-mostly (3.5, 2)	Mostly (4, 1)	Rarely-sometimes (2.5, 2)	Mostly (4, 1)
Visual refreshing	Mostly (4, 1.75)	Mostly (4, 2)	Mostly (4, 2)	Mostly (4, 1)

*Note.* For Q1 (overall strategy), 1 = *verbal strategy only*, 5 = *visual strategy only*; for all other questions, 1 = *never*, 5 = *always*

Mann-Whitney *U* tests were used to assess potential differences between young and older adults’ strategy use data, first within the control and then in the instructed condition. In the control group, there was a significant effect of age on: labelling, *U* = 343, *z* = −2.33 , *p* = .020 (mean rank, young = 37.78; mean rank, older = 27.22); automatically noticing semantics, *U* = 362, *z* = −2.07, *p* = .039 (mean rank, young = 37.19; mean rank, older = 27.81); active use of semantics, *U* = 363.50, *z* = −2.04 , *p* = .042 (mean rank, young = 37.14; mean rank, older = 27.86); and overall use of semantics, *U* = 363.50, *z* = −2.06 , *p* = .039 (mean rank, young = 37.14; mean rank, older = 27.86; all other *U* ≥ 431.50, all other *p* > .26). In the instructed group, there were no significant differences in strategy use between the two age groups, suggesting that a more similar strategy profile is associated with semantic instruction (all *U* ≥ 426.00, all *p* > .22).

Mann-Whitney *U* tests were also used to investigate potential differences in strategy use between the control and instruction conditions, first within the young adults and then in the older adults. In young adults, there were no significant effects of instruction on any of the strategy reports (all *U* ≥ 431.00, all *p* > .25). In older adults, there was a significant effect of instruction on: active use of semantics, *U* = 339.50, *z* = −2.38, *p* = .018 (mean rank, control = 27.11; mean rank, instructed = 37.89); and overall use of semantics, *U* = 331, *z* = −2.52, *p* = .012 (mean rank, control = 26.84; mean rank, instructed = 38.16; all other *U* ≥ 434.00, all other *p* > .27). This further suggests that older adults’ strategy profile was positively associated with semantic strategy instruction, in that those who received the instruction reported reliably more active and overall use of semantics than those in the control condition.

Finally, Spearman’s correlations assessed relationships among strategy reports and capacity in each task version (see Table 6). However, it is important to acknowledge that, due to the sample size (N = 32 in each correlation), estimates are likely not to have stabilized (Schönbrodt & Perugini, 2013). In terms of the emerging pattern of relationships, and first considering young adults, the control group’s capacity in the high semantic task was negatively associated with counting up the number of cells (i.e., more counting associated with lower performance). However, high semantic task performance was positively associated with overall use of semantics (both small-moderate correlations). There were no significant correlations when considering the instructed participants’ task performance. For older adults, both the control and instructed group’s capacity in the high semantic task was positively associated with active use of semantics, while the instructed group’s capacity in the high semantic task was also positively associated with overall use of semantics (all small-moderate correlations).

**Table 6:** Spearman’s correlations between reported strategy use and visual working memory performance across low and high semantic tasks, for young and older adults in control and instructed conditions in Experiment 2

			High semantic	Overall strategy	Combining	Counting up	Labelling	Automatic semantics	Active semantics	Overall semantics	Visual refreshing
Young adults	Control	Low semantic	.68***	.01	.01	-.23	.28	.18	.27	.28	-.03
		High semantic	-	.05	.08	-.36*	.31	.23	.27	.37*	-.20
	Instructed	Low semantic	.54**	.01	.14	-.16	.24	-.07	-.01	-.03	.23
		High semantic	-	-.18	.17	-.15	.02	-.23	.17	.19	.29
Older adults	Control	Low semantic	.73***	-.13	.20	-.16	.13	.35	.33	.19	-.25
		High semantic	-	.04	-.02	-.12	-.01	.27	.47**	.26	-.02
	Instructed	Low semantic	.79***	-.10	.06	.03	.19	.05	.22	.21	-.21
		High semantic	-	-.16	.02	.12	.19	.11	.35*	.36*	.03

*Note.* *p < .05; **p < .01; ***p < .001. N = 32 in each group. Correlations stabilize at *N* > 250 (Schönbrodt & Perugini, 2013)

### Discussion

Experiment 2 findings showed the expected age-related difference in visual working memory capacity (Swanson, 2017). They also highlighted a benefit of semantic availability in visual working memory, but for young adults only (Hamilton et al., 2018). The results showed no overall benefit of semantic strategy instruction, along with, importantly, no negative impact on performance. Furthermore, the variables did not interact to produce the predicted outcome that semantic strategy instruction would specifically boost older adults’ capacity in the more difficult, low semantic task.

The benefit of semantic availability for young, but not older, adults aligns with Hamilton et al. (2018), who also found a lack of benefit of higher semantic availability in a visual matrix task for older adults. However, it is important to acknowledge that Bayesian statistics showed only anecdotal evidence for this effect. Interestingly, Nicholls and English (2020) found that older adults did benefit from semantic availability within a visual matrix task, but only to the same extent as young adults, despite their lower overall capacity. Therefore, it seems that older adults are less reliably able to draw upon the cognitive resources involved in using and benefitting from higher semantic availability in this highly age-sensitive task (see also Forsberg et al., 2020b, for age-related differences in the benefit of verbal labels). Another important finding was the interaction between semantic availability and administration order. Regardless of age group, participants performed better on the high semantic task when it was performed last. Therefore, task practice is likely related to improved strategy development and implementation over time (Rowe et al., 2008).

Considering strategy use, some interesting effects were observed. In the control group, older adults tended to report a less efficient strategic approach in comparison to young adults, with less incorporation of labelling and semantic-based strategies (Nicholls & English, 2020). It is worth considering, however, that strategy selection itself may not be the sole factor that is important for older adults, but that they may require more time and task practice for them to effectively implement their strategic approach, according to task demands (Nicholls & English, 2020). Instruction did not affect young adults’ strategy use but appears to have encouraged more use of semantic strategies in older age.

There were also some positive relationships observed between strategy use and high semantic task performance in the control and instructed groups. Young adults’ performance was positively correlated with overall semantic strategy use under control conditions, and older adults’ performance was positively associated with active semantic strategy use across both control and instructed conditions. Furthermore, older adults’ high semantic task performance was positively associated with overall semantic strategy use in the instructed condition. While our sample size was too limited for these correlations to be considered stable, this evidence suggests that strategies are important to working memory performance. Promisingly, this could also suggest that strategy instruction can potentially encourage a more efficient strategic approach in older age. However, at least under these circumstances, this was only correlated with memory for more meaningful patterns, for which semantic codes are more easily identified. Importantly, research with larger samples must be conducted to yield more robust support for the correlational findings.

## General discussion

This research aimed to investigate the effect of semantic availability and strategy use during an age-sensitive visual matrix task in young and older adult age. We tested whether semantic strategy instruction could encourage participants to use efficient, semantic-based strategies, with the aim of boosting task capacity. Experiment 1 investigated these variables in young adults, measuring recognition. Experiment 2 additionally included older adults, measuring recall. Several key conclusions can be drawn. First, young adults appear to benefit from high semantic availability when measuring recall, but not recognition. Second, young adults demonstrate more efficient spontaneous strategy use compared with older adults. Third, and most importantly, at least under the present conditions, semantic strategy instruction did not benefit capacity for either age group. Considering modulation of reported strategy use, in Experiment 1, there was no significant difference in semantic strategy use between the young adult control and instructed groups. However, in Experiment 2, older adults in the instructed group reported more actively searching for meaningful shapes and using semantics overall. This provides some promising evidence regarding the possibility of instructing older adults in semantic strategies, to use their cognitive resources more efficiently during age-sensitive cognitive tasks. This is particularly important in the context of rapidly aging populations and the increasing number of people experiencing cognitive decline. However, instruction techniques will require further development and analysis.

### The role of semantics in visual working memory

Meaningful, 'high semantic' visual working memory tasks are typically easier than more abstract, 'low semantic' tasks, likely because of activation of semantic concepts in long-term memory (Brown & Wesley, 2013). Semantic codes can be automatically activated at perception or strategically created, which attaches meaning to otherwise abstract patterns (Riby & Orme, 2013). Ultimately, although high semantic stimuli may automatically activate semantic representations in long-term memory upon perception, evidence shows that the associated performance benefit appears to come at a cognitive cost. Executive resources appear to be required to form and/or rehearse meaningful representations in the context of the specific, abstract visual pattern (Brown & Wesley, 2013). In Experiment 1, capacity was numerically higher for high semantic patterns in the instructed group, suggesting a potential benefit of visual semantics; however, this did not meet significance, and was not supported by Bayesian analysis. It must be considered that most existing research has measured recall rather than recognition. Indeed, these paradigms include different cognitive processes, and the latter may be less sensitive to effects of semantics (Allen et al., 2018).

A key aim was to assess whether older adults could differentially benefit from semantic availability in the current task given their age-related deficit in capacity, as previous findings are inconsistent (e.g., Hamilton et al., 2018; Nicholls & English, 2020). Nicholls and English found that older adults were able to benefit from higher semantic availability within visual matrix tasks, but only to the same extent as young adults, despite having lower initial capacity. In contrast, Hamilton et al. (2018) showed less promising findings, demonstrating a lack of semantic benefit for older adults. Supporting findings by Hamilton et al., the present Experiment 2 showed that older adults were unable to benefit from the high semantic task, whereas there was anecdotal evidence that young adults were able to do so (see also Brown & Wesley, 2013; Orme et al., 2017; Riby & Orme, 2013). Therefore, older adults may be less able to reliably draw upon long-term memory semantics to scaffold their performance. It may be that limitations in attentional resources in older age and/or processing speed underlie the lack of benefit from high semantic stimuli within this age-sensitive task (Brown et al., 2012). Notably, semantic availability also interacted with administration order, showing a reliable semantic benefit when the high semantic task was performed last. Importantly, this cannot simply be explained by task practice. For example, numerically, young adults who performed the high semantic task first did not go on to perform better on the low semantic task (see OSM).

The findings do not necessarily mean that older adults are unable to benefit from semantic availability. The stimuli in the current task were abstract, black-and-white checkered patterns. Therefore, even the more meaningful, high semantic task was still relatively abstract. Perhaps visual semantics may be easier for older adults to meaningfully encode when using different stimuli such as larger grids which allow for even more meaningful representations, or stimuli which are more realistic and encountered more frequently in the real world. Furthermore, it is possible that older adults could have benefitted from semantic availability if they had received more task practice (Rowe et al., 2008) and/or extensive training in the strategy. For example, Forsberg et al. (2020a; see also Laine et al., 2018) showed some positive results from a visualization strategy instruction protocol for both young and older adults’ *n*-back task performance using a 30 min training session. Notably, even then, older adults appeared to have greater difficulty implementing the strategy and there was less overall benefit compared with young adults. Furthermore, previous studies have not always shown positive effects of strategy instruction paradigms even in young adults’ visual working memory (e.g., Bengson & Luck, 2016).

### Strategic approach

Another key aim of this research was to investigate strategies and the potential for strategy instruction to encourage participants to use a more efficient semantic strategy, aimed at boosting capacity. We have uniquely shown that use of semantics is a commonly reported spontaneous strategy in visual working memory. Importantly, there was no reliable effect of semantic strategy instruction on capacity.

The current study also observed age-related differences in strategy use during this age-sensitive cognitive task (Lemaire, 2016). Older adults reported spontaneously relying on more obvious visual-based strategies and incorporated verbal labelling and semantic strategies less (Nicholls & English, 2020). This supports a number of studies that have shown that young adults display a larger strategy repertoire, which is more flexible according to task demands, in a range of cognitive tasks. For example, measuring recall for visual matrix tasks, Nicholls and English found that older adults displayed a less varied and flexible spontaneous strategic approach compared to young adults. Furthermore, older adults have been found to be less likely than young adults to spontaneously adopt effective strategies to study paired associates (Dunlosky & Hertzog, 2001). In studies investigating how young and older adults solve arithmetic problems, across groups, young and older adults both reported a strategy repertoire comprising nine strategies. However, when counting the number of strategies used by each individual, young adults used an average of five strategies and older adults used only three (Hodzik & Lemaire, 2011; Lemaire & Arnaud, 2008). Limitations in executive resources could account for age-related differences in strategic approach (Reuter-Lorenz & Lustig, 2016).

Attempts should now be made to further understand the complexities of strategy use across the adult lifespan. In the current experiments, the strategy questionnaire was administered after task completion to probe typical, spontaneous strategy use, and to ensure that the questions did not influence this. However, this is a limitation of this research, as it is unknown what trials participants were drawing upon to complete the questionnaire. Indeed, research has shown that participants may use different combinations of strategies for the same task on different trials (Morrison et al., 2016). In future work, strategy use could be assessed throughout task performance, for example on a trial-by-trial or block-by-block basis, gaining data about variation in strategy use over time (Lemaire, 2016). Importantly, this could also be helpful when measuring potential differential impacts of strategy instructions with age (e.g., Atkinson et al., 2018). Another limitation of this research was the use of a Likert scale-based strategy questionnaire. It is unknown how participants were interpreting questions or response options and if young and older adults were interpreting these similarly. Research could also include richer, free-text responding to broaden our understanding about the development and implementation of strategies (Ozimič et al. 2023).

### Semantic strategy instruction

Promisingly, it appears that instruction may have encouraged older adults to use semantic strategies to a greater extent, but the benefit of these strategies was limited. Indeed, this is in line with previous strategy training studies which have found a limited effect of instructing semantic-based strategies (Bailey et al., 2009; Bartsch et al., 2019; Bartsch & Oberauer, 2019, 2021; McNamara & Scott, 2001; Turley-Ames & Whitfield, 2003).

Potentially, to observe a reliable effect of instruction on capacity, a more comprehensive training protocol may be needed, such as incorporating extensive training with more examples of how to use a semantic strategy, and greater practice. Furthermore, research may need to take initial capacity into account, as strategy instruction may not be implementable by individuals with low capacity and may even hinder performance in some instances (Nyberg et al., 2003).

## Conclusions

In conclusion, this research aimed to investigate the impact of semantic availability and semantic strategy instruction on visual working memory task performance in young and older age. The findings highlight age-related differences in visual working memory and support evidence suggesting that older adults are less able to take advantage of semantic availability to scaffold visual working memory. This could be related to limitations in central executive resources in older age, which are likely required to take advantage of the benefit of high semantic stimuli. There was an interesting pattern of findings regarding strategic approach across groups. Spontaneously (i.e., in the control group), young adults demonstrated incorporating verbal-based strategies like labelling and semantic strategies to a greater extent than older adults. However, semantic strategy instruction may have encouraged greater use of semantic strategies in older age, despite not boosting overall capacity experimentally. It is now necessary to test more extensive training protocols, for example using more thorough instructions and task practice prior to experimental task completion. This may demonstrate a more robust effect of semantic strategy instruction on young and/or older adults’ visual working memory capacity.

## Supplementary information

Below is the link to the electronic supplementary material.

Supplementary file1 (DOCX 43 KB)

## Data Availability

Both experiments were pre-registered (https://osf.io/aemb7/). Data and materials underlying this study are available via the Open Science Framework (https://osf.io/aemb7/; https://osf.io/fg3rc/).

## Footnotes

1 Note, as per the study pre-registration, we also conducted a G* Power analysis using the ‘ANOVA: Repeated-measures, within-between interaction’ statistical test. This indicated a required sample size of 34 participants to observe an interaction with a medium effect size (*f* = .25; *α* = .05; power (1 – β) = .95; correlation among repeated measures = .7, based on low versus high semantic task capacity in young adults under control conditions (Nicholls & English, 2020)). We have since discovered that this analysis may underestimate the required sample size (Brysbaert, 2019). Nevertheless, the target sample size was ultimately driven by the power needed for paired *t*-tests. A G*Power sensitivity analysis shows that, with N = 44, we were powered (1 – β = .8) to detect effect size *f* = .43 (*α* = .05; numerator df = 1; number of groups = 2). Furthermore, this sample size is on par with that used in similar published work (e.g., Allen et al., 2021).

2 Note, previous studies have commonly generated lures by solely changing the location of a black cell (method 1). However, this effectively changes two cells (i.e., changes a white cell to black and changes a black cell to white). Therefore, in conjunction with piloting, we used the three lure generation methods to ensure we avoided a ceiling effect. Furthermore, the equal probability of these three approaches ensured that participants could not only rely on knowledge of the number of filled/unfilled cells to complete the task.

3 Note also that the direction of the response scale for all but questionnaire item 1 is presently reversed relative to these earlier papers, for ease of interpretation (i.e., higher score = greater strategy use).

## Code availability

Analysis syntax underlying this study is available via the Open Science Framework (https://osf.io/aemb7/).
