# Supplementary material for: A semantic strategy instruction intervention aimed at boosting young and older adults’ visual working memory capacity
Source: Mem Cognit. 2025 Mar 5;53(6):1677–95. doi: 10.3758/s13421-024-01676-8 (PMC12402024; doi:10.3758/s13421-024-01676-8)
Supplement: Supplementary file 1 — Supplementary file1 (DOCX 43 KB) [file 13421_2024_1676_MOESM1_ESM.docx]

**A semantic strategy instruction intervention aimed at boosting young and older adults’ visual working memory capacity**

**Rebecca Hart & Louise A. Brown Nicholls**

**Supplementary File**

**Experiment 1**

***Strategy instructions- control***

These are examples of the kinds of patterns you will be asked to remember. These patterns are quite small in size and are taken from the beginning level of the task. As the task continues the pattern sizes will increase.

In each trial, try to take in the first pattern that is presented while it is in view, and try to remember it during the delay period. Then, respond to the second pattern that appears, to indicate whether or not you think it is the same as the first. The next trial will then involve a new pattern to remember, and so on throughout the task.

When approaching this task, it is possible to use a variety of strategies. At the end of the memory task, we will ask you to report the extent to which you feel you used different strategies.

Note, you will be asked to carry out the task two times in total, with a number of trials within each task version. The second task will essentially be the same as the first, but with new patterns to remember. Each task version will take approximately 10-15 minutes.

***Strategy instructions- instructed***

These are examples of the kinds of patterns you will be asked to remember. These patterns are quite small in size and are taken from the beginning level of the task. As the task continues the pattern sizes will increase.

In each trial, try to take in the first pattern that is presented while it is in view, and try to remember it during the delay period. Then, respond to the second pattern that appears, to indicate whether or not you think it is the same as the first. The next trial will then involve a new pattern to remember, and so on throughout the task.

When approaching this task, it is possible to use a variety of strategies. At the end of the memory task, we will ask you to report the extent to which you feel you used different strategies.

One way to support performance of this task is to activate and use any meaning or familiarity contained within the patterns, such as letters, symbols, or even everyday objects or animals. For example, in the pattern on the left above, you may notice that the black cells resemble a letter ‘T’ on its side. As the patterns increase in size and complexity, the patterns may even resemble more complex shapes, such as animals or everyday objects. You can try to use that knowledge to help you remember what the patterns looks like. Even if you don’t notice anything meaningful relatively automatically or straight away, you could try to search for meaning. For example, the pattern on the right above may not be as obviously meaningful, but if you look you may notice that some of the white cells resemble a back-to-front ‘L’, or the black cells at the bottom could together resemble the shape of a dog. Please try your best to implement this strategy of finding meaning in the patterns. You may use this strategy alongside any other strategies you find useful, but do try to use this strategy as much as possible throughout the task.

Note, you will be asked to carry out the task two times in total, with a number of trials within each task version. The second task will essentially be the same as the first, but with new patterns to remember. Each task version will take approximately 10-15 minutes.

***Strategy Questionnaire***

Thank you for participating in this study. We very much appreciate your time and effort. Before finishing, we would be grateful if you could answer some questions regarding how you feel you went about the task today. There are a variety of possible ways to try to remember the patterns. For example, you may have found you tended to rely on visual strategies only, on verbal strategies only, on visually meaningful information (i.e. familiarity), or some combination of these approaches. Please respond to the below questions to indicate how, on average, you approached the task:

1. In this task overall, please indicate the extent to which you relied upon a visual and/or verbal strategy to help you remember a pattern. A visual strategy involves concentrating on your mental image of what the pattern looks like. A verbal strategy involves verbalising (mentally or out loud) the features of the pattern and concentrating on that verbal information. Please select the response that best represents your approach:

| “I used a verbal strategy only” | “I used mostly verbal but some visual rehearsal” | “I used verbal and visual strategies equally” | “I used mostly visual but some verbal rehearsal” | “I used a visual strategy only” |
| --- | --- | --- | --- | --- |

1. How often did you *combine* visual and verbal strategies to help you remember a pattern?

| “Never” | “Rarely” | “Sometimes” | “Most of the time” | “Always” |
| --- | --- | --- | --- | --- |

1. How often did you “count up” the number of black or white cells?

| “Never” | “Rarely” | “Sometimes” | “Most of the time” | “Always” |
| --- | --- | --- | --- | --- |

1. How often did you use verbal labels to rehearse a pattern (e.g., naming a collection of cells the letter “L” and repeating “L” mentally or out loud)?

| “Never” | “Rarely” | “Sometimes” | “Most of the time” | “Always” |
| --- | --- | --- | --- | --- |

1. How often did you notice meaningful or familiar shapes within a pattern, without trying (e.g. automatically noticing that a collection of cells resembles a symbol, animal, etc.)?

| “Never” | “Rarely” | “Sometimes” | “Most of the time” | “Always” |
| --- | --- | --- | --- | --- |

1. How often did you actively try to find meaningful or familiar shapes within a pattern (i.e. looking for collections of cells that might resemble symbols, animals, etc.)?

| “Never” | “Rarely” | “Sometimes” | “Most of the time” | “Always” |
| --- | --- | --- | --- | --- |

1. Regardless of how you initially noticed meaningful or familiar shapes, how often did you use that meaningful or familiar information to remember a pattern (e.g. remembering that a collection of cells resembled symbols, animals, etc.)?

| “Never” | “Rarely” | “Sometimes” | “Most of the time” | “Always” |
| --- | --- | --- | --- | --- |

1. How often did you focus upon refreshing your mental image of the pattern?

| “Never” | “Rarely” | “Sometimes” | “Most of the time” | “Always” |
| --- | --- | --- | --- | --- |

***Pattern size analyses***

A mixed Analysis of Variance (ANOVA) was carried out to investigate effects of strategy instruction (control, instructed; between participants), semantic availability (low, high; repeated measures), and pattern size (small (Levels 4-7), medium (Levels 8-11), large (Levels 12-15); repeated measures) on accuracy (see Table S1). There were no significant effects of strategy instruction, *F*(1,42) = .19, *MSE* = .039, *p* = .667, η^2^_p_ = .004, *BF*_incl_ = .28, or semantic availability, *F*(1,42) = 2.01, *MSE* = .013, *p* = .164, η^2^_p_ = .046, *BF*_incl_ = .34 However, there was a significant effect of pattern size, *F*(2,84) = 56.06, *MSE* = .014, *p* < .001, η^2^_p_ = .572, *BF*_incl_ = 1.58 x 10^13^. Follow-up, Bonferroni-corrected paired *t*-tests (to meet significance, *p* ≤ .0166) showed that accuracy for small patterns (*M* = .93; *SD* = .08) was superior to that for medium (*M* = .81; *SD* = .12), *t*(43) = 7.28, *p* < .001, *BF*_10_ = 2.40 x 10^6^, and large patterns (*M* = .74; *SD* = .11), *t*(43) = 11.53, *p* < .001, *BF*_10_ = 7.23 x 10^11^. Memory was also better for the medium than the large patterns, *t*(43) = 3.39, *p* = .001, *BF*_10_ = 20.76. There were no significant interactions (all *F* < 1.43, all *p* > .24, all *BF*_incl_ < .48).

The same mixed ANOVA was carried out on the response time (RT) data. Where sphericity could not be assumed, Greenhouse-Geisser correction was used. Again, only the effect of pattern size was significant, *F*(1.7,72.2) = 18.39, *MSE* = 1192375, *p* < .001, η^2^_p_ = .305, *BF*_incl_ = 25948 (all other *F* < .63, all other *p* > .43, all other *BF*_incl_ < .31). Bonferroni-corrected paired *t*-tests (to meet significance, *p* ≤ .0166) showed that RT for small patterns (*M* = 1827; *SD* = 759) was quicker than for medium (*M* = 2439; *SD* = 1058), *t*(43) = -5.12, *p* < .001, *BF*_10_ = 2786, and large patterns (*M* = 2734; *SD* = 1368), *t*(43) = -5.26, *p* < .001, *BF*_10_ = 4329. However, there was no reliable difference between medium and large patterns, *t*(43) = -1.88, *p* = .067, *BF*_10_ = .82.

**Table S1**

*Mean accuracy scores (proportion correct) and response times (ms; both with SDs) for the control and instructed groups by semantic task version and pattern size in Experiment 1.*

|  |  | Low Semantic | | | High Semantic | | |
| --- | --- | --- | --- | --- | --- | --- | --- |
|  |  | Small | Medium | Large | Small | Medium | Large |
| Accuracy | Control | .92  (.08) | .81  (.15) | .73  (.13) | .93  (.09) | .82  (.16) | .73  (.14) |
|  | Instructed | .91  (.14) | .82  (.15) | .72  (.14) | .95  (.08) | .80  (.16) | .80  (.13) |
| Response Time | Control | 1851  (1058) | 2322  (945) | 2624 (1352) | 1919 (892) | 2469 (1046) | 2943 (2310) |
|  | Instructed | 1827  (963) | 2494  (1585) | 2551 (1052) | 1712  (832) | 2472  (1385) | 2820 (1692) |

***Administration order analyses***

An exploratory 2 (semantic strategy instruction: control, instructed) x 2 (administration order: low semantic first, high semantic first) x 2 (semantic availability: low, high) mixed-factorial ANOVA was run to test the potential effect of administration order on both accuracy and RT (Nicholls & English, 2020). There were no significant effects or interactions involving administration order (all *F* < 2.24, all *p* > .14, all *BF*incl < .75).

***Correlation analyses***

Spearman’s correlation analyses were carried out to assess relationships amongst strategy reports and performance for both groups (see Table S2). A significant, positive correlation was found between the instructed group’s accuracy in the low semantic task and the use of semantics. Another significant finding was a negative correlation between the control group’s accuracy in the low semantic task and verbal labelling (i.e., greater labelling associated with lower accuracy). There was also a significant, positive correlation between the control group’s accuracy in the high semantic task and visual refreshing. However, due to the very limited sample size, these correlations will not be sufficiently stabilised to determine meaningful relationships (Schönbrodt et al. 2013).

**Table S2**

*Spearman’s correlations between reported strategy use and accuracy data in control and instructed groups in Experiment 1 (N = 22 in each group).*

|  |  | High Accuracy | Overall Strategy | Combining | Counting Up | Labelling | Automatic Semantics | Active Semantics | Use of Semantics | Visual Refreshing |
| --- | --- | --- | --- | --- | --- | --- | --- | --- | --- | --- |
| Control | Low Accuracy | .37 | -.10 | .31 | .12 | -.50* | -.06 | -.16 | -.33 | .31 |
|  | High Accuracy | - | -.00 | .33 | .14 | .01 | .33 | -.05 | .07 | .50* |
| Instructed | Low Accuracy | .44* | -.21 | .34 | -.09 | -.01 | .06 | .10 | .53* | .27 |
|  | High Accuracy | - | -.02 | .05 | -.22 | .16 | .39 | .33 | .24 | .09 |

*Note.* * *p* < .05*; ** p < .01; ***p < .001.*

**Experiment 2**

***Strategy instructions - control***

These are examples of the kinds of patterns you will be asked to remember. These patterns are quite small in size and are taken from the practice level of the task. As the task continues the pattern sizes will increase.

In each trial, try to take in the first pattern that is presented while it is in view, and try to remember it during the delay period. Then, try to recall it using the paper templates. The next trial will then involve a new pattern to remember, and so on throughout the task.

When approaching this task, it is possible to use a variety of strategies. At the end of the memory task, we will ask you to report the extent to which you feel you used different strategies.

Note, you will be asked to carry out the task two times in total, with a number of trials within each task version. The second task will essentially be the same as the first, but with new patterns to remember.

***Strategy instructions - instructed***

These are examples of the kinds of patterns you will be asked to remember. These patterns are quite small in size and are taken from the practice level of the task. As the task continues the pattern sizes will increase.

In each trial, try to take in the first pattern that is presented while it is in view, and try to remember it during the delay period. Then, try to recall it using the paper templates. The next trial will then involve a new pattern to remember, and so on throughout the task.

When approaching this task, it is possible to use a variety of strategies. At the end of the memory task, we will ask you to report the extent to which you feel you used different strategies.

One way to support performance of this task is to activate and use any meaning or familiarity contained within the patterns, such as letters, symbols, or even everyday objects or animals. For example, in the pattern on the left above, you may notice that the black cells resemble a letter ‘T’ on its side. As the patterns increase in size and complexity, the patterns may even resemble more complex shapes, such as animals or everyday objects. You can try to use that knowledge to help you remember what the patterns looks like. Even if you don’t notice anything meaningful relatively automatically or straight away, you could try to search for meaning. For example, the pattern on the right above may not be as obviously meaningful, but if you look you may notice that some of the white cells resemble a back-to-front ‘L’, or the black cells at the bottom could together resemble the shape of a dog. Please try your best to implement this strategy of finding meaning in the patterns. You may use this strategy alongside any other strategies you find useful, but do try to use this strategy as much as possible throughout the task.

Note, you will be asked to carry out the task two times in total, with a number of trials within each task version. The second task will essentially be the same as the first, but with new patterns to remember.

***Administration order data***

**Table S3**

*Young and older adults’ mean capacity (span) scores (± SDs) from Experiment 2, for low and high semantic tasks by instruction group and administration order.*

|  |  |  | Low Semantic | High Semantic |
| --- | --- | --- | --- | --- |
| Young adults | Control | Low first | 8.56 (1.38) | 9.27 (1.19) |
|  |  | High first | 8.98 (1.53) | 9.06 (2.25) |
|  | Instructed | Low first | 8.46 (1.60) | 9.42 (2.15) |
|  |  | High first | 8.98 (1.88) | 8.98 (1.89) |
| Older adults | Control | Low first | 6.83 (1.61) | 7.15 (2.19) |
|  |  | High first | 6.60 (1.46) | 5.69 (1.85) |
|  | Instructed | Low first | 6.77 (1.66) | 7.12 (1.71) |
|  |  | High first | 6.75 (2.00) | 6.54 (1.89) |
